# Supplementary figures and images for: Metabolic modulation regulates cardiac wall morphogenesis in zebrafish
Source: eLife. 2019 Dec 23;8:e50161. doi: 10.7554/eLife.50161 (PMC7000217; doi:10.7554/eLife.50161)

**Figure 2—source data 1 – Mass spectrometry data.**


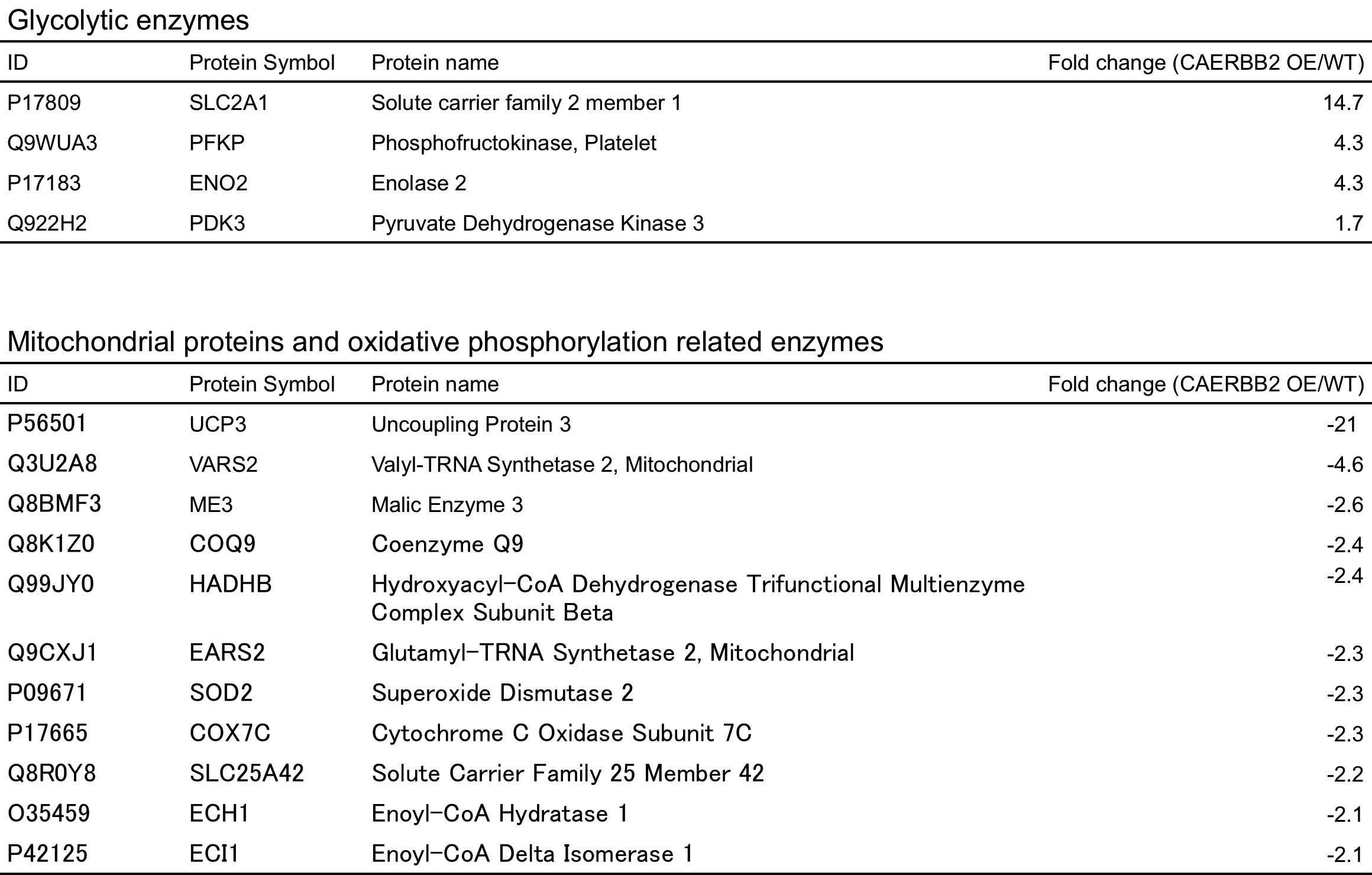

Supplement: Figure 2—source data 1. — P7 WT and CAERBB2 OE mouse hearts were isolated and protein expression levels analyzed by mass spectrometry. All presented proteins are statistically significant at p<0.05. [file elife-50161-fig2-data1.docx]

**Figure 2—source data 2 – Primer sequences for qPCR analysis.**


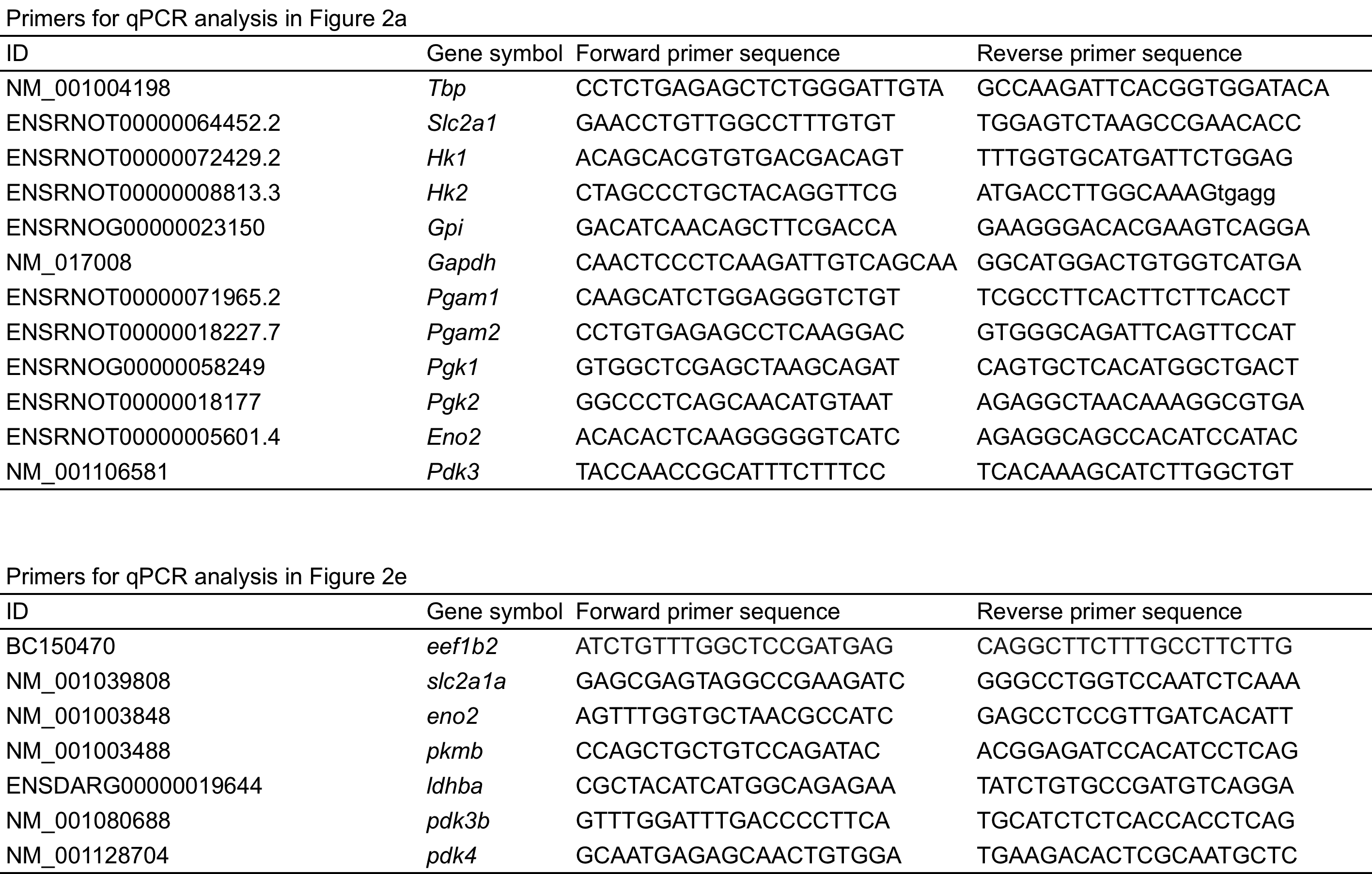

Supplement: Figure 2—source data 2. — Primer sequences used in Figure 2a and e. [file elife-50161-fig2-data2.docx]

**Figure 2—source data 3 – Mean Ct values of qPCR analysis in Figures 2a and 2e.**


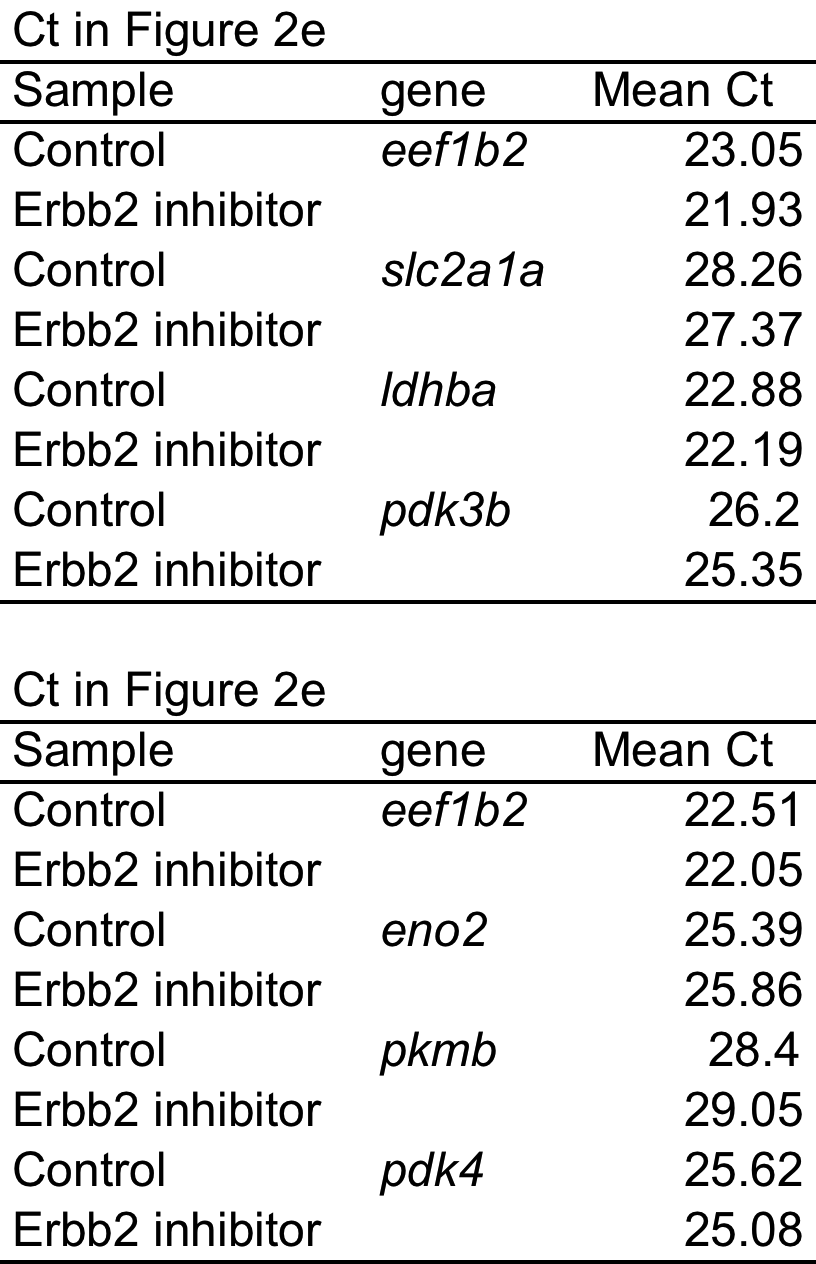

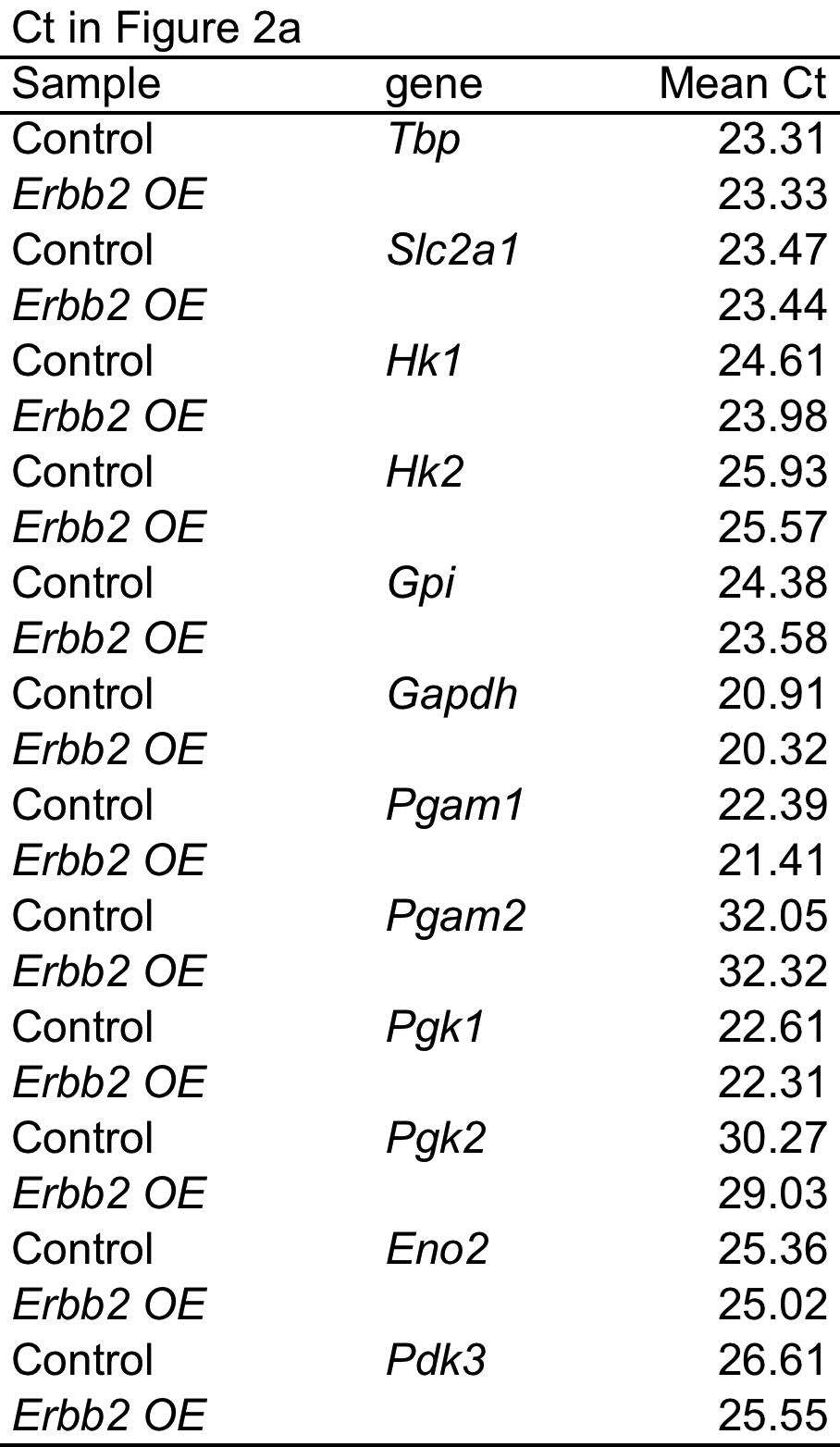

Supplement: Figure 2—source data 3. [file elife-50161-fig2-data3.docx]
